# User-Centered Delivery of AI-Powered Health Care Technologies in Clinical Settings: Mixed Methods Case Study

**DOI:** 10.2196/76241

**Published:** 2025-08-26

**Authors:** Meredith Schreier, Randall Brandt, Hien Brown, T Saensuksopa, Christine Silva, Laura M Vardoulakis

**Affiliations:** 1 Google (United States), 1600 Amphitheater Parkway, Mountain View, CA, 94043, United States, 1 6502530000; 2 Mile Bluff Medical Center, Mauston, United States; 3 MEDITECH, Canton, MA, United States

**Keywords:** user-centered research, software design, delivery of healthcare, implementation science, electronic health record

## Abstract

**Background:**

Providers spend a large percentage of their day using electronic health record (EHR) technology and frequently report frustration when EHR tasks are time-consuming and effortful. To solve these challenges, artificial intelligence (AI)–based enhancements to EHR technology are increasingly being deployed. However, AI-based implementations for EHR features often lack user-centered evaluation.

**Objective:**

This study evaluates, using a user-centered approach, the implementation of an AI-powered search and clinical discovery tool within an EHR system.

**Methods:**

We conducted a mixed methods study consisting of interviews, observations, and surveys for 5 months.

**Results:**

High adoption rates for the AI-based features (163/176, 93% users after 3 months) and significant increases across key metrics, including user satisfaction (U=49; *P*<.001) and perception of time saved (U=49; *P*<.001), demonstrated that the AI-based features were not only successfully integrated into various clinical workflows but also improved the user experience for clinicians.

**Conclusions:**

Our results underscore the feasibility and effectiveness of using a user-centered approach for the deployment of clinical AI tools. High adoption rates and positive user experiences were driven by our user-centered research program, which emphasized close collaboration with users, rapid incorporation of feedback, and tailored user training. This study program can be used as a starting framework for the design and integration of human-centered research methods for AI tool deployment in clinical settings.

## Introduction

Artificial intelligence (AI)–based tools have permeated culture and industry, generating excitement, hesitation, and visions of opportunity [1-3]. This is especially true in health care: AI-based tools have transformative potential, despite known challenges spanning implementation, security, infrastructure, user experience, and product performance [4-7]. While electronic health records (EHRs) have been transformative in moving health care professionals to a more digital and connected way of working, challenges of use can lead to increased time spent using the system, increased feelings of frustration, and general dissatisfaction among users [8-14]. As EHR systems incorporate AI-based features into their products, questions remain about whether these solutions will improve the clinical user experience.

The incorporation of human-centered research methods, including the engagement of end users throughout the development process, is critical to the development of usable products that meet clinical users’ needs [15,16] and has a long history within the field of human-computer interaction [17,18]. Previous research has demonstrated the value and feasibility of user-centered research methods during the development of AI-based clinical tools [19-21]. In this paper, we build upon previous research by demonstrating how user-centered evaluations can extend to the evaluation of clinical AI tools during the final stages of implementation.

Through this research, we sought to understand if an AI-based search and summarization tool [22], previously used side by side with an EHR, could be successfully integrated directly within the EHR. To test the implementation approach and measure the experiential impact on users at a health care system, we developed a multitouchpoint user-centered study program that emphasized strong partnerships, close collaboration with users, and robust evaluations focused on newly unlocked product capabilities. The research methods draw from long-established practices in user experience design and research [23-25], as well as newer guidelines for AI-based research [26], which were adapted for use within the clinical environment.

**Figure 1.**
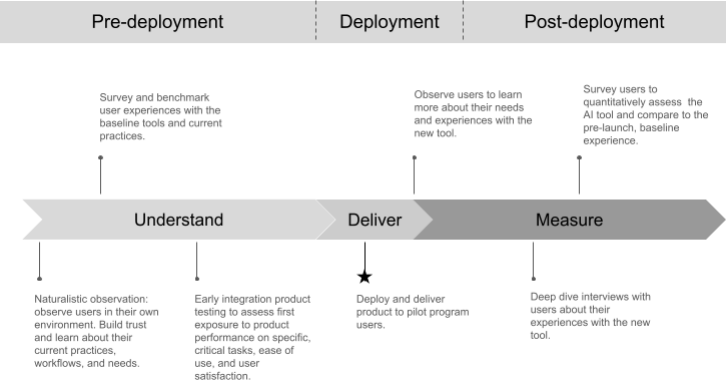
User-centered study program timeline. AI: artificial intelligence.

This paper makes two primary contributions. First, we introduce a collection of research activities that form a novel, user-centered framework for implementing and evaluating AI-based clinical tools (Figure 1). This framework emphasizes the importance of conducting both qualitative and quantitative methods to evaluate the user experience and collecting signals from users throughout multiple stages of implementation and deployment. Further, there is flexibility in the specific methods that can be used in a collection such as this, making it an adaptable means of conducting human-centered research.

**Figure 2.**
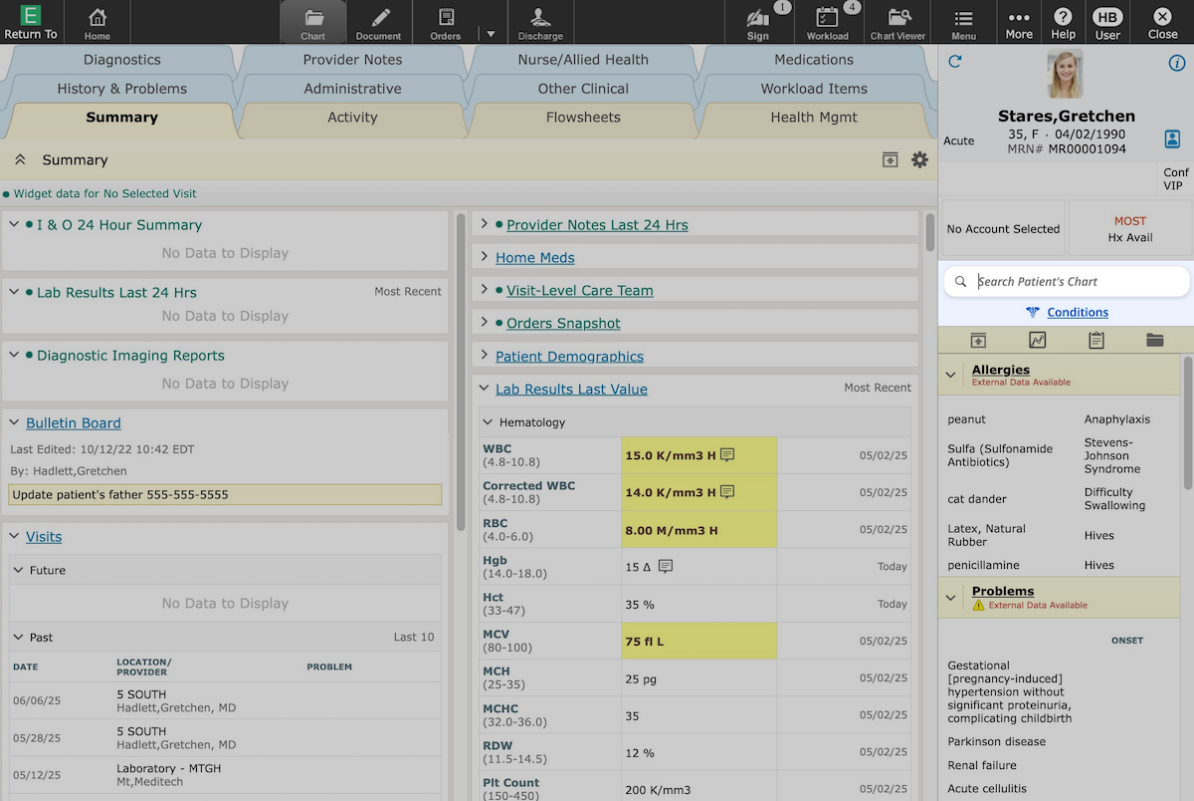
Artificial intelligence (AI)–powered search integration within MEDITECH’s Expanse, highlighted. All data shown in this figure are synthetic (ie, realistic but not real) patient data. Participants in the study interacted with real patient data.

Second, we apply this framework through a case study of the delivery of Expanse Navigator (Google and MEDITECH; Figures2  3; Multimedia Appendix 1), an AI-powered search and summarization tool accessible within MEDITECH’s Expanse EHR. Through this case study, we show both the feasibility of the approach and how it enabled rapid incorporation of insights into the delivery strategy, including factors influencing use, potential risks, and user satisfaction.

**Figure 3.**
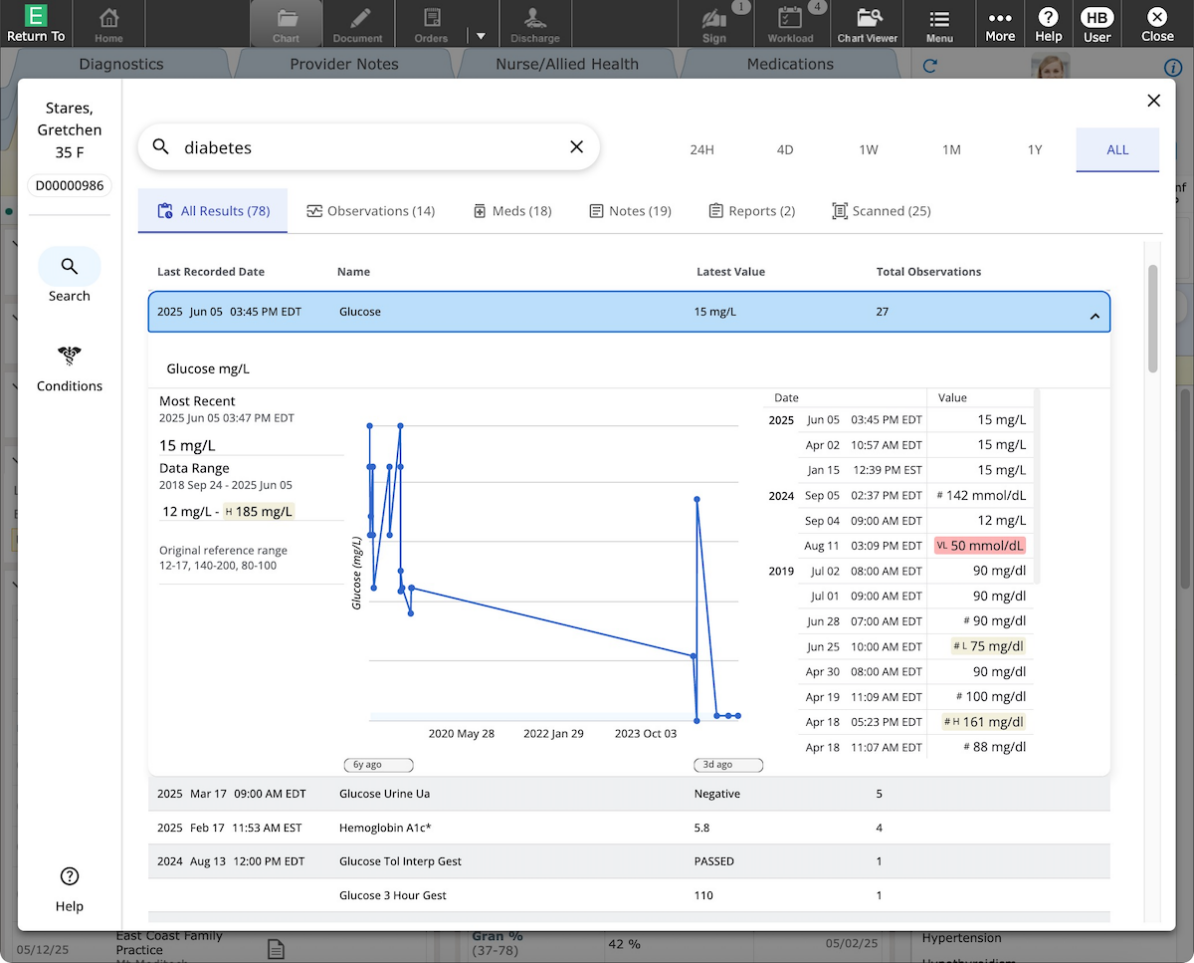
Search results experience. All data shown in this figure are synthetic (ie, realistic but not real) patient data. Participants in the study interacted with real patient data.

## Methods

### Overview

A multistep, mixed methods, user-centered study was developed and run for 5 months. The goal of this research program was to measure the qualitative and quantitative experiential effects of an AI-based search and summarization tool (referred to as Expanse Navigator) within MEDITECH’s Expanse and to understand the feasibility of incorporating user-centered methods during the implementation phases of AI-based clinical tools.

### Program Site and Participants

Mile Bluff Medical Center is a 40-bed acute-care hospital and outpatient care center in Mauston, Wisconsin, United States. A total of 176 health care professionals were provided access to a clinical pilot of Expanse Navigator in January 2024; this includes 53 medical doctors or advanced practice providers, 61 registered nurses, 48 allied health professionals, and 14 health information management specialists across 24 distinct clinical and operational departments.

All pilot participants were invited to participate in the user-centered research program. A sample of 128 out of the 176 (72%) pilot program members opted into the program.

### Materials

Expanse Navigator offers advanced search capabilities and an AI-generated, comprehensive view of a patient’s health history (Figures2  3; Multimedia Appendix 1). It works together with and is accessible directly within MEDITECH’s Expanse. The tool offers new capabilities, unlocks valuable data (eg, PDFs and faxes), and makes them quickly and easily accessible to users via a modern search experience. The search capabilities handle misspellings and abbreviations, provide suggested search terms, and give results ranked by relevance. The tool is powered by AI rather than keyword matching.

### Naturalistic Observation

The first component of the user-centered program was clinical observation. Before the deployment of Expanse Navigator, 21 representative users from the hospital, ambulatory care, outpatient clinics, and the emergency department were observed in their normal clinical environments (outside of patient care). Observations provided an understanding of users’ existing tools, workflows, environments, and experiences before the deployment of Expanse Navigator. Further, this effort helped to establish collaboration and build rapport with clinical users.

### Survey Program

The second component of the user-centered program focused on experiential measurement through the use of pre- and postdeployment surveys. To develop the survey measures, MEDITECH and Google jointly developed a set of primary metrics: user satisfaction, product helpfulness across specified tasks (Multimedia Appendix 2), and perceived time savings. Survey questions used fully labeled 5-point scales, following industry best practices [27], and are available in the Multimedia Appendix 3. Survey questions were optional; respondents were not required to answer all questions.

Before deploying Expanse Navigator, we measured prelaunch metrics of MEDITECH’s Expanse via a baseline survey. Four weeks following deployment, we distributed a postlaunch survey to collect identical metrics on users’ experiences with Expanse Navigator.

Surveys were administered to all pilot program participants. There were 94 out of 176 (73%) responses and 73 out of 176 (57%) responses to the baseline survey and postlaunch survey, respectively.

### Early Integration Product Testing

The third component of the user-centered program involved early integration of product testing. The purpose of this assessment was to test the integrated product’s ability to support key clinical tasks, to collect feedback based on users’ first exposure to the product, and to ensure that no issues that could prevent easy and efficient use were present ahead of widespread launch.

During a single 60-minute session, participants viewed 3 minutes of prerecorded onboarding content and had 5 minutes of unstructured exploration with the product. Immediately thereafter, participants were prompted with 12 structured tasks to complete using Expanse Navigator features (Multimedia Appendix 4). Tasks were scored as “completed,” “completed with difficulty,” or “not completed.” The tasks were informed by findings from naturalistic observation sessions and designed to reflect responsibilities fulfilled in the course of daily work. Sessions were conducted until data confidently showed that users could complete key tasks. In our case, this research phase ended after sessions with 3 users showed high levels of success. This methodology facilitated unique feedback related to intuitiveness and ease of use and also highlighted areas of focus for user onboarding and education.

### User Interviews

The final component of the user-centered program was qualitative interviews. Approximately 6 weeks after launching Expanse Navigator, we conducted 60-minute interviews with 4 users. We sought to capture qualitative data around the impact of the product, users’ experiences, high-priority use cases, and self-reported engagement. Participants spanned distinct roles and specialties and had not previously participated in one-on-one user-centered research sessions.

### Data Analysis

Completed survey responses were analyzed using descriptive statistics (eg, percentage of users reporting satisfaction, perceived helpfulness, and time savings). To measure the experiential impact of the AI tool, a within-subjects Mann-Whitney *U* test (1-tailed) was run on each metric, using data from respondents who completed both baseline and postlaunch surveys. Qualitative interviews were analyzed by conducting a thematic analysis of detailed interview notes [28].

### Ethical Considerations

This research was reviewed by the Advarra institutional review board and determined to be exempt from IRB oversight. All participants provided informed consent before participating.

## Results

Early integration testing demonstrated that users completed tasks with relative ease, having received minimal instructions, and no major obstacles to efficient and easy use were observed, clearing the way for further deployment. Results from this research stage highlighted the need to explicitly onboard users to the new structure and categories by which information was organized.

Postdeployment, our 3 primary metrics for Expanse Navigator, described above in the “Introduction” section and Figures2  3, saw strong ratings from users (Table 1): 62 out of 72 (86%) respondents reported high satisfaction, 45 out of 60 (75%) respondents described high helpfulness, and 63 out of 69 (91%) respondents reported that Expanse Navigator felt faster than the baseline system.

**Table 1.** Experiential ratings of the artificial intelligence (AI)–based experience from participants who completed the postlaunch survey: primary metric scores as percentages.

Metric	Postlaunch survey, n (%)
Satisfaction (N=72)	
Users who reported being “somewhat” or “very” satisfied	62 (86)
Helpfulness (N=60)	
Average % of users who rated the product as “very” or “extremely” helpful in accomplishing the 7 specific tasks	45 (75)
Perceived time savings (N=69)	
Users who rated the system as “somewhat” or “much” faster than the previous system	63 (91)

Notably, when compared to baseline results, users rated the AI-based experience more favorably across all metrics (Tables 2-4). Qualitative analyses showed that clinicians had use for the product at all points of care, identified the key value derived, and provided data to inform potential future features.

**Table 2.** Within-subjects comparison of ratings among participants who completed both baseline and postlaunch surveys: primary metric scores as percentages.

Metric	Baseline survey, n (%)	Postlaunch survey, n (%)
Satisfaction, N=19		
Users who reported being “somewhat” or “very” satisfied	2 (11)	15 (79)
Helpfulness, N=15		
Users who rated the product as “very” or “extremely” helpful in accomplishing the 7 specific tasks averaged across tasks on average	2 (13)	8 (53)
Perception of time savings, N=18		
Users who rated the system as “somewhat” or “much” faster than the previous system	9 (50)	17 (94)

**Table 3.** Within-subjects comparison of user satisfaction and perceived time savings: 1-tailed Mann-Whitney *U* test results. Effect size is given by the rank biserial correlation.

Mann-Whitney *U* test	U	*P* value	Rank-biserial correlation (*r*)	SE of rank-biserial correlation
Satisfaction score	49.00	<.001	−0.729	0.188
Time savings score	74.50	.002	−0.540	0.193

**Table 4.** Within-subjects comparison of helpfulness: 1-tailed Mann-Whitney *U* test results. Effect size is given by the rank biserial correlation.

Helpfulness scores per task	U	*P* value	Rank-biserial correlation (*r*)	SE of rank-biserial correlation
Task 1	35.00	<.001	−0.689	0.211
Task 2	68.00	.03	−0.396	0.211
Task 3	46.50	.003	−0.587	0.211
Task 4	32.00	<.001	−0.716	0.211
Task 5	88.00	.15	−0.218	0.211
Task 6	73.00	.08	−0.305	0.215
Helpfulness task #7	51.50	.009	−0.510	0.215

Results from the program at large demonstrated that the AI-based experience was highly adaptable across various clinical and operational settings. In addition, the user-centered approach provided the clinical and AI teams with rapid data on the tool’s effectiveness and identified valuable focus areas for user training. This data-driven approach, paired with positive word-of-mouth and user experiences, fueled rapid delivery and adoption. When measured 3 months post rollout, 163 out of 176 (93%) participants had accessed Expanse Navigator, and over 4000 searches had been performed.

## Discussion

### Principal Findings

The results of this study not only demonstrate that the AI-powered search and clinical discovery tool led to significant improvements in user satisfaction, helpfulness, and perception of time saved, but also demonstrate the feasibility of evaluating the clinical experience through a mixed method, user-centered approach.

AI-based tools are becoming increasingly common in clinical settings. As these technologies continue to advance, so will user expectations for high-quality product experiences. It will become increasingly important to measure the user experience of these tools with robust approaches that can be flexibly applied within a clinical environment. This research program was intentionally designed to evaluate product experience, refine understanding of user mental models, and optimize our onboarding program for the greatest impact when deploying an AI tool.

Incorporating user-centered research during predeployment stages allowed for the collection of benchmark measures of user satisfaction, helpfulness, and speed. It also informed the design of onboarding experiences and custom training for cohorts with specialized workflows. Furthermore, the activities during this stage led to a strong partnership with hospital leadership, which, in turn, increased awareness about the upcoming product launch.

The deployment and postdeployment research allowed the team to measure the impact of the AI tool from a clinical user’s point of view. The results of our case study demonstrate that the AI-based search and summarization experience, accessible directly within an EHR, provides users with new capabilities and increased levels of satisfaction, helpfulness, and speed.

Both the lift and feasibility of a user-centered approach were demonstrated through strong user participation, survey completion rates, and the overall duration of data collection (60 minutes per one-on-one study session).

The design of this study program follows previous work showing the importance of assessing the user experience during all phases of the product development life cycle: design, development, and deployment [29,30], and addresses calls for an increase in human-centered evaluative research alongside AI clinical deployments [31]. It extends past research that outlines approaches to user-centered research during design and development stages of AI-based clinical tools [19-21], and provides a framework for assessments that can occur during the later stages of clinical implementation.

### Limitations

There are several limitations to this research. First, while the research involved a large percentage of users for the AI-based tool within MEDITECH’s Expanse, across a wide range of clinical roles and specialities, it did so with users from a single medical center. Though we demonstrate feasibility for the user-centered study program presented in this paper, future research is needed to demonstrate generalizability. Second, while the findings from the case study show positive improvements in the user experience for this AI-based EHR integration, further research would be needed to demonstrate the impact across additional clinical measures.

### Conclusions

By developing and executing a mixed methods, user-centered program for AI tool deployment, we enabled trust, collaboration, and frequent communication between clinical end users and AI practitioners, and a robust collection of key product metrics. This data-driven and user-centered approach championed end users’ experiences and surfaced those findings pre- and postproduct launch, highlighting specific areas for curated user training and optimizing the opportunity for rapid deployment. Integrating user-centered research as part of the deployment process ensures that AI tools are not only technologically advanced but are also relevant, usable, useful, and safe for use in any setting; notably, all of these benefits are of critical importance for tools used in health care and clinical settings.

Prioritizing a human-centered research program for AI-based tool deployment supports effective product integration and user satisfaction; further, it supports the thoughtful delivery of products and services in high-stakes health care environments where patient safety is the utmost priority.

## Supplementary material

10.2196/76241 Multimedia Appendix 1 Summarization experience. All data shown in this figure are synthetic (ie, realistic but not real) patient data. Participants in the study interacted with real patient data.

10.2196/76241 Multimedia Appendix 2 Survey program: helpfulness tasks.

10.2196/76241 Multimedia Appendix 3 Survey measures. Prelaunch surveys assessed baseline experiences with “Search Patient’s Chart,” and postlaunch surveys assessed experiences with “Search and Summarization.”

10.2196/76241 Multimedia Appendix 4 Early integration testing task set.

## Abbreviations

AI: artificial intelligence
EHR: electronic health record
