# Supplementary figures and images for: User-Centered Delivery of AI-Powered Health Care Technologies in Clinical Settings: Mixed Methods Case Study
Source: JMIR Hum Factors. 2025 Aug 26;12:e76241. doi: 10.2196/76241 (PMC12380366; doi:10.2196/76241)

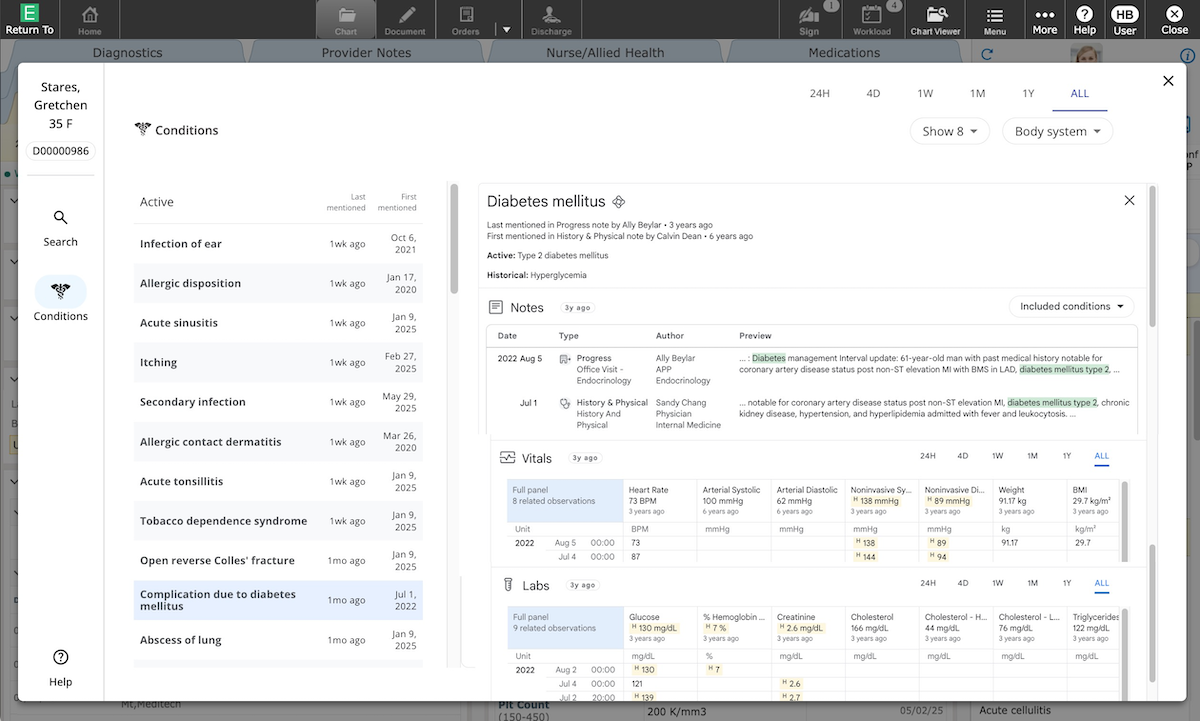

Supplement: Multimedia Appendix 1 [file humanfactors-v12-e76241-s001.png]
