# Supplementary material for: User-Centered Delivery of AI-Powered Health Care Technologies in Clinical Settings: Mixed Methods Case Study
Source: JMIR Hum Factors. 2025 Aug 26;12:e76241. doi: 10.2196/76241 (PMC12380366; doi:10.2196/76241)
Supplement: Multimedia Appendix 2 [file humanfactors-v12-e76241-s002.pdf]

Multimedia Appendix 2: [Survey Program - Helpfulness Tasks]

Survey program helpfulness tasks

In the past two weeks, how helpful was the tool for	
**Task 01**	Finding a specific scanned document (e.g., consent form)
**Task 02**	Finding a specific diagnostic report (e.g., imaging report)
**Task 03**	Finding a specific report from a different healthcare organisation
**Task 04**	Finding information from notes (including handwritten documents)
**Task 05**	For looking for a specific lab test (e.g., CBC)
**Task 06**	Finding the result from a specific report (e.g., MRI)
**Task 07**	For answering targeted questions about a patient
