# Supplementary material for: User-Centered Delivery of AI-Powered Health Care Technologies in Clinical Settings: Mixed Methods Case Study
Source: JMIR Hum Factors. 2025 Aug 26;12:e76241. doi: 10.2196/76241 (PMC12380366; doi:10.2196/76241)
Supplement: Multimedia Appendix 3 [file humanfactors-v12-e76241-s003.pdf]

Multimedia Appendix 3: [Pre- and Post-launch Survey Measures]

Survey measures. Pre-launch surveys assessed baseline experiences with “Search Patient’s Chart” and post-launch surveys assessed experiences with “Search and Summarization".

**Satisfaction**	Overall, how **satisfied** are you with [Search Patient’s Chart / Search and Summarization] ?	Single select:<br><br>● Very satisfied<br>● Somewhat satisfied<br>● Neither satisfied nor dissatisfied<br>● Somewhat dissatisfied<br>● Very dissatisfied
**Helpfulness**	Overall, how **helpful** is [Search Patient’s Chart / Search and Summarization] ?	Single select:<br><br>● Extremely helpful<br>● Very helpful<br>● Moderately helpful<br>● Slightly helpful<br>● Not at all helpful
**Helpfulness**	In the past two weeks, how **helpful** was [Search Patient’s Chart / Search and Summarization] for achieving the following?<br><br>rows<br>● Finding a specific scanned document (e.g. consent form)<br>● Finding a specific diagnostic report (e.g. imaging report)<br>● Finding a specific report from a different healthcare organization<br>● Finding information from notes (including handwritten documents)<br>● Looking for a specific lab test (e.g. CBC)<br>● Finding the result from a specific report (e.g. MRI)<br>● Answering targeted questions about a patient<br>● Other (specify)	Single select columns:<br><br>● Extremely helpful<br>● Very helpful<br>● Moderately helpful<br>● Slightly helpful<br>● Not at all helpful<br>● Did not use for this purpose
**Time (self-perceived)**	How, if at all, does your **use** of [Search Patient’s Chart / Search and Summarization] **change the amount of time** needed to find important patient information?<br>Complete the following sentence.<br>**Using** [Search Patient’s Chart / Search and Summarization] **makes finding information _____**	Single select:<br><br>● Much faster than before<br>● Somewhat faster than before<br>● Neither faster nor slower than before<br>● Somewhat slower than before<br>● Much slower than before
