# Supplementary material for: User-Centered Delivery of AI-Powered Health Care Technologies in Clinical Settings: Mixed Methods Case Study
Source: JMIR Hum Factors. 2025 Aug 26;12:e76241. doi: 10.2196/76241 (PMC12380366; doi:10.2196/76241)
Supplement: Multimedia Appendix 4 [file humanfactors-v12-e76241-s004.pdf]

Multimedia Appendix 4: [Early Integration Testing - Task Set]

Early integration testing task set

Patient A	Patient B
**Task 01**: Find and open Patient A's record	**Task 07**: Find and open Patient B's record
**Task 02**: Find Patient A's most recent *(condition-based)* lab value	**Task 08**: Find the reason for Patient B's consult with an outside system
**Task 03**: Find Patient A's progress note from 02/2022	**Task 09**: Share the findings from Patient B's most recent MRI
**Task 04**: Find and share 3 of Patient A's active conditions	**Task 10**: Share Patient B's most recent sodium level
**Task 05**: Determine and share which physician first diagnosed the patient with (*condition*)	**Task 11**: Share when Patient B had a (*specific operation*)
**Task 06**: Find the procedure Patient A had in March 2022 and review the corresponding anaesthesia record.	**Task 12**: Share Patient B's (*condition-specific*) medication dosage as of July 2023
